# Ankle osteoarthritis: an online survey of current treatment practices of UK-based podiatrists and physiotherapists

**DOI:** 10.1186/s13047-023-00683-3

**Published:** 2023-12-12

**Authors:** Michael J. Callaghan, Jinal Pravin Gala, Edward Roddy

**Affiliations:** 1 Department of Health Professions, Manchester Metropolitan University, Manchester, UK; 2 Centre for Musculoskeletal Research, University of Manchester, Manchester, UK; 3 Department of Physiotherapy, Manchester University Hospitals NHS Foundation Trust, Manchester, UK; 4 School of Medicine, Keele University, Staffordshire, UK

**Keywords:** Ankle, Osteoarthritis, Physiotherapy, Podiatry, Survey

## Abstract

**Background:**

Patients with painful ankle osteoarthritis (OA) have a mixed experience of non-surgical management which they may attribute to a lack of guidance for clinicians on usual care treatment. Therefore, the purpose of this study was to survey the current clinical practices of UK-based physiotherapists and podiatrists for the treatment of painful ankle osteoarthritis (OA).

**Methods:**

UK-based physiotherapists and podiatrists who treat patients with ankle OA completed a self-administered online questionnaire about their professional and clinical service characteristics, diagnostic criteria, treatment aims, preferred treatment options, and treatment outcome measures. Data were collected anonymously and stored on JISC online survey. ‘Usual care’ was defined as a combination of ‘Always’, and ‘Frequently’, and ‘Not usual care’ was defined as ‘Sometimes’ ‘Rarely,’ ‘Never’, and ‘not applicable’ combined. Statistically significant differences in responses between the physiotherapists and podiatrists were analysed using X^2^ tests for each treatment modality. Statistical significance was set at *p* < 0.05.

**Results:**

Between 1st June 2021 and 31st August 2021, 100 responses were received; 2 were invalid. Of the 98 valid responses, 63 were from physiotherapists and 35 from podiatrists. The most common treatment aims in both professions were to reduce pain (*n* = 87, 89%) and improve quality of life (*n* = 82, 84%). 50 respondents (51%) offered 3 or 4 treatment sessions and 53 respondents (54%) saw patients for 30–40 min at the first treatment session.

The five most common modalities used by physiotherapists were patient education (*n* = 63, 100%), teaching self-management (*n* = 58, 92%), lifestyle modification (*n* = 54, 86%), ankle strengthening (*n* = 55, 87%), and proprioception exercises (*n* = 54, 86%). For podiatrists, these were patient education (*n* = 35, 100%), ankle strengthening (*n* = 31, 89%), activity pacing (*n* = 28, 80%), lifestyle modification (*n* = 27, 77%), and gait training (*n* = 27, 77%).

**Conclusions:**

This first-ever survey revealed physiotherapists’ and podiatrists’ current practices to treat painful ankle OA. This study provides a better understanding of how ankle OA is treated in UK current clinical practice and can inform future clinical trials to compare current practice with new treatment modalities.

## Background

Ankle pain has been estimated to account for around 3% of primary care consultations in adults aged over 50 years with those aged 71–80 years placing the greatest burden on GPs [1]. The population prevalence of symptomatic radiographic ankle osteoarthritis (OA) in the over 50 s is 3.4% [2]. Post traumatic (PT) OA is the predominant form of OA in the ankle, accounting for 70% of cases, followed by rheumatoid arthritis (12%) and primary/idiopathic OA (7%) [3].

Ankle OA is highly problematic with severe pain being the most disabling symptom, resulting in a substantial negative impact on a person’s physical and mental wellbeing [4]. Many people with ankle OA described their pain in emotive terms such as ‘terrible’ and ‘horrendous’ [4]. It is known that mental and physical disability from ankle OA is at least as severe as in those with end-stage hip OA [4].

International clinical guidelines recommend non-pharmacological interventions such as exercises and weight loss as first-line management for OA, regardless of joint site [5, 6]. In the UK, physiotherapists and podiatrists provide non-surgical treatment for ankle OA within the NHS or in private practice. Whereas several evidenced-based non-surgical management options for clinicians and patients are available for other joints [7], specific evidence for ankle OA interventions is sparse and people with ankle OA report mixed experiences of non-surgical management [4]. Extrapolating recommendations from other joints may not be appropriate and there is a lack of guidance for clinicians on treatment best practices [4]. We undertook a survey of UK-based physiotherapists and podiatrists to understand better how painful ankle OA is managed in current clinical practice.

## Methods

### Design

This was a cross-sectional online survey. Approval was obtained from the research ethics committee of Manchester Metropolitan University (33838).

### Participants

UK-based registered physiotherapists and podiatrists registered with the Health Care Professions Council (HCPC) were asked to complete a self-administered online questionnaire. Participants were required to care for people with symptomatic ankle OA in their clinical practice but there were no exclusions for age, length of experience, or clinical setting. Those not practising in the UK or not wishing to give consent were excluded. Professional interactive networks, social media (Twitter) and the interactive Chartered Society of Physiotherapy (iCSP) message board were used to advertise the survey to UK-based physiotherapists with a target population of approximately 14,000. Podiatrists were approached online via the website of the Royal College of Podiatrists (https://rcpod.org.uk), which has a target population of 1119, and interactive networks such as podiatryarena.com (https://podiatryarena.com/index.php) and its Facebook (https://www.facebook.com/podiatryarena/) and Twitter pages (PodArena; (https://twitter.com/PodArena).

### Procedures

The participant information sheet and the consent form were on the first page of the online survey. Participants’ written consent to participate was not requested as informed consent was implied through voluntary completion of the survey.

### Questionnaire development

The questionnaire was developed by the authors and then presented to and piloted by five physiotherapists and podiatrists (at least 15 years qualified) who were experienced in managing ankle OA. They were asked to review the survey, refine the format and question design, to comment and provide feedback on the content of the questions (including treatment modalities listed), online presentation, the look and appeal of the survey, ease of completion, and relevance to clinical practice.

### Questionnaire content

Participants were advised that for the purpose of this survey ankle OA was defined as talocrural (tibiotalar) joint OA and did not include the subtalar joint. Screening questions ensured all participants were working in the UK and treated patients with ankle OA in their usual clinical practice. The survey was set up on the JISC platform (www.onlinesurvey.ac.uk) and consisted of 5 parts: I) participant characteristics, II) clinical service characteristics and diagnostic criteria, III) treatment aims, IV) preferred treatment options, and V) treatment outcome measures. Most questions were closed-ended multiple-choice questions, with an option for open-ended answers in parts II to V. The questionnaire also asked participants about their choices for diagnostic tests, treatment aims, and outcome measures.

### Data analysis

Data were collected anonymously and stored on JISC online survey, transferred and saved on a passcode-secured Excel sheet, and backed up on the university server. Data were analysed with IBM® SPSS® Statistics 26 (IBM Corp., Armonk, NY). Descriptive statistics of counts and proportions for categorical variables were calculated and displayed using histograms and tables. We combined the response options of ‘always’, and ‘frequently’, to indicate what both professions commonly used as treatment options and ‘sometimes’ ‘rarely’, ‘never’, and ‘not applicable’ for treatments not commonly used. Differences in responses between the physiotherapists and podiatrists were analysed using Chi squared tests for each treatment modality. Statistical significance was set at *p* < 0.05.

## Results

Between 1^st^ June to 31^st^ August 2021, there were 506 views on the survey homepage, from which 100 responses were received. Two were invalid due to one respondent not being a UK resident and another not managing people with ankle OA. Of the 98 valid responses, 63 (64%) were from physiotherapists and 35 (36%) from podiatrists; 80 (82%) practised in England. Forty-nine (50%) of the respondents worked in the NHS, 34 (35%) in private practice, and nine (9%) in both the private and NHS sectors. The highest qualification was a diploma for 15 respondents (15%), a bachelor’s degree for 37 (38%), a master’s level qualification for 42 (43%) and a PhD for four (4%). Fifty respondents (51%) offered 3 or 4 treatment sessions and 53 respondents (54%) saw patients for 30–40 min at the first treatment session. Fifty (51%) of respondents treated one patient per week with ankle OA, 24% saw four or more patients (Table 1). Referrals were most commonly from general practitioners (*n* = 69, 70%) and orthopaedic surgeons (*n* = 66, 67%) (Table 1). Just under half the respondents (49%) worked in a multi-disciplinary team.

**Table 1:** Clinical service characteristics and diagnostic criteria, treatment aims and outcome measures for ankle OA

QUESTIONS	RESPONSE OPTIONS	TOTAL N (%)
Number of patients seen weekly with ankle OA	1	50 (51)
	2	16 (16)
	3	8 (9)
	4	6 (6)
	5	4 (4)
	> 5	13 (14)
Source of ankle OA referrals*	GENERAL PRACTITIONER	69 (70)
	ORTHOPAEDIC SURGEON	66 (67)
	SELF-REFERRAL/ PATIENT REFERRAL	39 (40)
	RHEUMATOLOGIST	37 (38)
	PHYSIOTHERAPIST	30 (31)
	PODIATRIST	25 (26)
	CHIROPRACTOR/ OSTEOPATH	9 (9)
	OTHER	4 (4)
Duration of the first treatment session	10 MINUTES	2 (2)
	20 MINUTES	6 (6)
	30 MINUTES	20 (20)
	40 MINUTES	37 (38)
	50 MINUTES	9 (9)
	60 MINUTES	22 (23)
	> 60 MINUTES	2 (2)
Number of treatment sessions including the first	1	7 (7)
	2	13 (13)
	3	26 (27)
	4	23 (24)
	5	12 (12)
	6	9 (9)
	> 6	8 (8)
Do you work as a part of a multi-disciplinary team while managing ankle OA?	YES	48 (49)
	NO	50 (51)
To which other healthcare practitioners do you refer individuals with ankle OA?*	ORTHOPAEDIC SURGEON	78 (80)
	RHEUMATOLOGIST	23 (24)
	PHYSIOTHERAPIST	46 (47)
	PODIATRIST	55 (56)
	ORTHOTIST	39 (40)
	OTHER	11 (11)
Features used to diagnose ankle OA*	AGE	66 (67)
	PAIN ON PALPATION	60 (61)
	SITE OF PAIN (ANKLE JOINT LINE)	81 (83)
	EARLY MORNING PAIN	63 (64)
	JOINT STIFFNESS RELIEVED ON MOVEMENT	78 (80)
	GENERAL SIGNS AND SYMPTOMS OF INFLAMMATION (WARMTH, REDNESS, SWELLING, ETC.)	63 (64)
	CREPITUS	62 (63)
	PAIN ON WEIGHT BEARING	76 (78)
	REDUCED ANKLE RANGE OF MOTION	90 (92)
	OVERWEIGHT	39 (40)
	HISTORY OF TRAUMA	71 (73)
	ACTIVITY STATUS (HISTORY/ PAST)	39 (40)
	IMAGING DIAGNOSIS (PLAIN X-RAY, CT SCANS, MRI, US)	85 (87)
	PAIN RELIEF AFTER LOCAL ANAESTHETIC INJECTION	22 (22)
	NO TEST/CRITERIA	1 (1)
	OTHER	6 (6)
Treatment aims*	REDUCE PAIN	87 (89)
	IMPROVE ANKLE STABILITY	59 (60)
	IMPROVE RANGE OF MOTION	60 (61)
	IMPROVE STRENGTH	65 (66)
	IMPROVE PROPRIOCEPTION	69 (60)
	IMPROVE ACTIVITIES OF DAILY LIFE (BATHING, GROOMING, FEEDING, SHOPPING, AMBULATION, DOING LAUNDRY)	71 (72)
	IMPROVE CARDIOVASCULAR/ AEROBIC FITNESS	28 (29)
	IMPROVE QUALITY OF LIFE	82 (84)
	SLOW DOWN THE PROGRESSION OF OA	36 (37)
	PREPARE FOR SURGERY	16 (16)
	TEACH SELF-MANAGEMENT	81 (83)
	OTHERS	2 (2)
Outcome measures*	NONE	7 (7)
	VISUAL ANALOGUE SCALE for PAIN	51 (52)
	NUMERICAL RATING SCALE for PAIN	42 (43)
	QUALITY OF LIFE QUESTIONNAIRE SCORE (SHORT FORM 36/12/6D, ETC.)	28 (29)
	ANKLE OA SCALE (FOOT AND ANKLE OUTCOME SCORE (FAOS), AMERICAN ACADEMY of ORTHOPEDIC SURGEONS FOOT AND ANKLE QUESTIONNAIRE, ETC.)	13 (13)
	WESTERN ONTARIO AND MCMASTER UNIVERSITIES OSTEOARTHRITIS (WOMAC) QUESTIONNAIRE	2 (2)
	PATIENT SATISFACTION	62 (63)
	GLOBAL RATING OF CHANGE SCORE (GROC)	4 (4)
	OTHERS	23 n

* Multiple answers could be selected for these questions therefore total responses may exceed 100%

Pain reduction (*n* = 87, 89%) and improving quality of life (*n* = 82, 84%) were the most frequent treatment aims. Several outcome measures were used, most commonly patient satisfaction (*n* = 62, 63%) or a visual analogue scale (*n* = 51, 52%) or a numerical rating scale (*n* = 42, 43%) to measure pain. Physiotherapists and podiatrists relied upon various features to diagnose ankle OA, the three most common were reduced ankle range of motion (*n* = 90, 92%), the site of pain (*n* = 81, 83%), and imaging (*n* = 85, 87%) (Table 1).

**Fig. 1:**
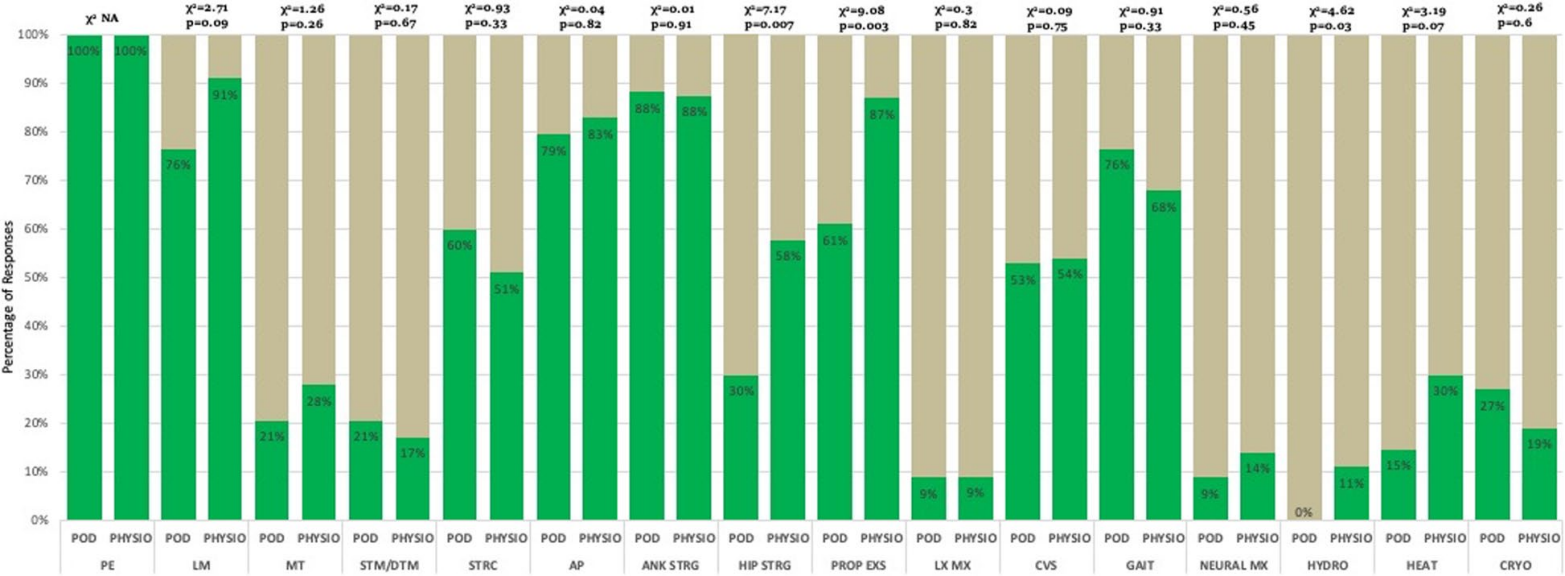
Treatment options for usual care and not usual care by physiotherapists and podiatrists. Figures are presented as a % of valid responses (*n* = 98). PHYSIO, physiotherapist; POD, Podiatrist; PE, patient education; LM, lifestyle modification; MT, manual therapy of peripheral joints; STM/DTM, soft tissue mobilisation/ deep tissue mobilisation; STRC, stretching; AP, activity pacing; ANK STRG, ankle strengthening; HIP STRG, hip strengthening; PROP EXS; proprioception exercises; LX, lumbar spine management; GAIT, gait management; NEURAL MX, neural mobilisation; HYDRO, hydrotherapy; HEAT, heating/ heat therapy; CRYO, cryotherapy. Χ.^2^- Pearson Chi-Square value and *p*- value of significance *P* < 0.05

**Fig. 2:**
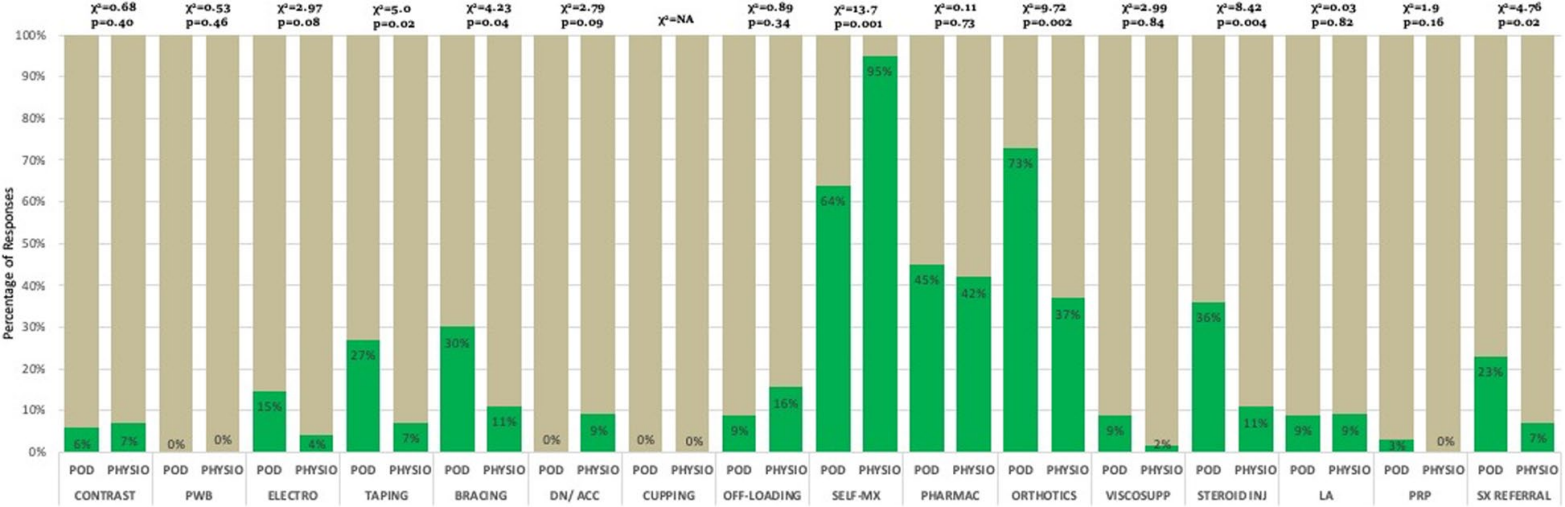
Treatment options for usual care and not usual care by physiotherapists and podiatrists. Figures are presented as a % of valid responses (*n* = 98). PHYSIO, Physiotherapist; POD, Podiatrist; CONTRAST, contrast bath; PWB, paraffin wax bath; ELECTRO, electrotherapy; DN/ACC, dry needling/ acupuncture; OFFLOAD, offloading; SELF MX, self-management; PHARMAC, pharmacotherapy; VISCOSUPP, visco-supplementation; Steroid Inj, steroid injection; LA, local anaesthesia injection; PRP, platelet-rich plasma therapy; SX, refer for surgery. χ.^2^- Pearson Chi-Square value and *p*-value level of significance *p* < 0.05

**Fig. 3:**
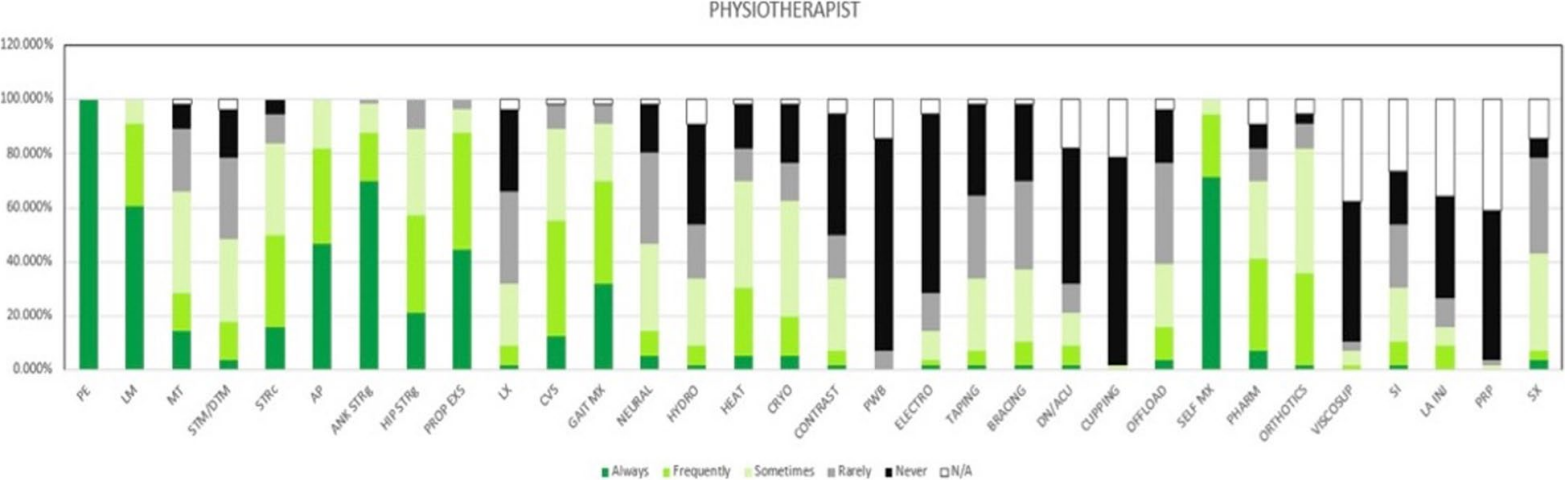
Treatment used for ankle OA by Physiotherapists. Figures are presented as a % of valid responses (*n* = 98). PE, patient education; LM, lifestyle modification; MT, manual therapy of peripheral joints; STM/DTM, soft tissue mobilisation/ deep tissue mobilisation; STRc, stretching; AP, activity pacing; ANK STRg, ankle strengthening; HIP STRg, hip strengthening; PROP EXS, proprioception exercises; LX, lumbar spine management; GAIT Mx, gait management; NEURAL, neural mobilisation; HYDRO, hydrotherapy; HEAT, heating/ heat therapy; CRYO, cryotherapy; CONTRAST, contrast bath; PWB, paraffin wax bath; ELECTRO, electrotherapy; DN/ACU, dry needling/ acupuncture; OFFLOAD, offloading; SELF MX, self-management; PHARM, pharmacotherapy; VISCOSUP, visco-supplementation; SI, steroid injection; LA INJ, local anaesthesia injection; PRP, platelet-rich plasma injection; SX, refer for surgery

**Fig. 4:**
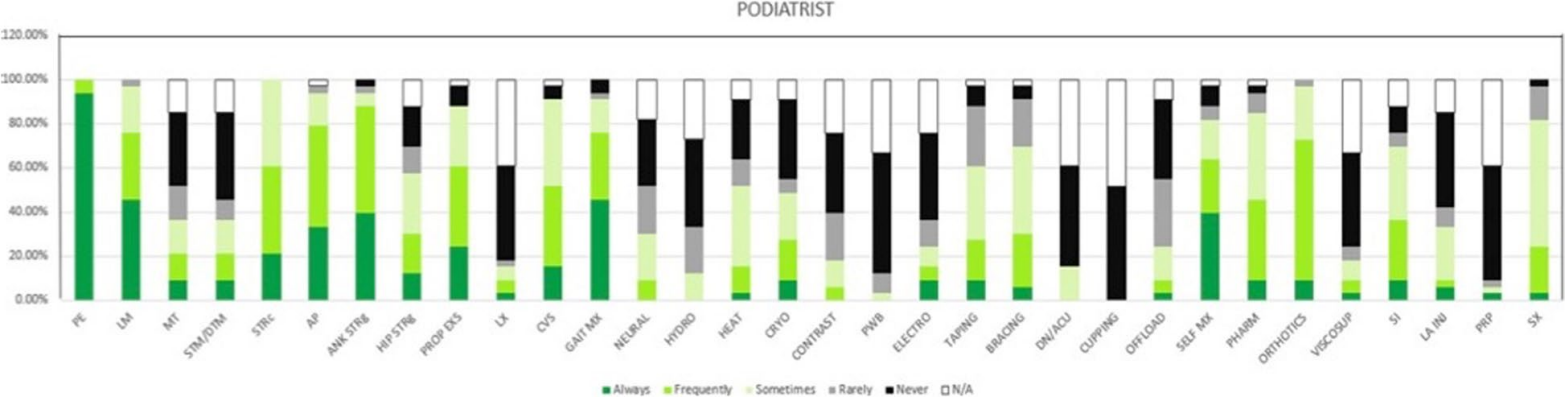
Treatment used for ankle OA by Podiatrists. Figures are presented as a % of valid responses (*n* = 98). PE, patient education; LM, lifestyle modification; MT, manual therapy of peripheral joints; STM/DTM, soft tissue mobilisation/ deep tissue mobilisation; STRc, stretching; AP, activity pacing; ANK STRg, ankle strengthening; HIP STRg, hip strengthening; PROP EXS, proprioception exercises; LX, lumbar spine management; GAIT Mx, gait management; NEURAL, neural mobilisation; HYDRO, hydrotherapy; HEAT, heating/heat therapy; CRYO, cryotherapy; CONTRAST, contrast bath; PWB, paraffin wax bath; ELECTRO, electrotherapy; DN/ACU, dry needling/ acupuncture; OFFLOAD, offloading; SELF MX, self-management; PHARM, pharmacotherapy; VISCOSUP, visco-supplementation; SI, steroid injection; LA INJ, local anaesthesia injection; PRP, platelet-rich plasma injection; SX, refer for surgery

The five most common modalities used by physiotherapists were patient education (*n* = 63, 100%), teaching self-management (*n* = 58, 92%), lifestyle modification (*n* = 54, 86%), ankle strengthening (*n* = 55, 87%), and proprioception exercises (*n* = 54, 86%) (Figs. 1, 2 & 3 and Table 2). For podiatrists, these were patient education (*n* = 35, 100%), ankle strengthening (*n* = 31, 89%), activity pacing (*n* = 28, 80%), lifestyle modification (*n* = 27, 77%), and gait training (*n* = 27, 77%) (Figs. 1, 2 & 4 and Table 2). Less than 30% of respondents in either professions opted for ankle bracing or taping as usual care. Physiotherapists used the treatment options of proprioception (*p* = 0.003), hip strengthening (*p* = 0.007), hydrotherapy (*p* = 0.031), self-management (*p* = 0.001) significantly more often than podiatrists. Taping (*p* = 0.025), bracing (*p* = 0.04), orthotics (*p* = 0.002), referral for surgery (*p* = 0.029), and corticosteroid injections (*p* = 0.004) were used significantly more often by podiatrists than physiotherapists.

**Table 2:** Specific treatments used for ankle OA by physiotherapists and podiatrists

TREATMENTS	RESPONSE OPTIONS	RESPONSE (%) PHYSIOTHERAPISTS	RESPONSE (%) PODIATRISTS	TREATMENTS	RESPONSE OPTIONS	RESPONSE (%) PHYSIOTHERAPISTS	RESPONSE (%) PODIATRISTS
PATIENT EDUCATION (*N* = 98)	Always Frequently Sometimes Rarely Never N/A	100% 0% 0% 0% 0% 0%	93.9% 6.1% 0% 0% 0% 0%	NEURAL MOBILITY (*N* = 98)	Always Frequently Sometimes Rarely Never N/A	5.3% 8.7% 31.6% 33.3% 17.5% 3.5%	0.% 9.1% 21.2% 21.2% 30.3% 18.9%
LIFESTYLE MODIFICATION (*N* = 98)	Always Frequently Sometimes Rarely Never N/A	61.4% 29.8% 8.8% 0% 0% 0%	45.5% 30.3% 21.2% 3% 0% 0%	HYDROTHERAPY (*N* = 98)	Always Frequently Sometimes Rarely Never N/A	1.8% 8.8% 24.6% 19.2% 36.8% 8.8%	0% 0% 12.1% 21.2% 39.4% 27.3%
MANUAL THERAPY-PERIPHERAL JOINT MOBILISATION (*N* = 98)	Always Frequently Sometimes Rarely Never N/A	14% 14% 36.8% 22.8% 10.5% 1.9%	9.1% 12.1% 15.2% 15.2% 33.3% 15.2%	HEAT (*N* = 98)	Always Frequently Sometimes Rarely Never N/A	5.3% 24.5% 40.3% 12.3% 15.8% 1.8%	3% 12.1% 36.4% 12.1% 27.3% 9.1%
SOFT TISSUE MOBILISATION/ DEEP TISSUE MOBILISATION (*N* = 98)	Always Frequently Sometimes Rarely Never N/A	3.5% 14% 29.8% 29.8% 17.5% 5.4%	9.1% 12.1% 15.1% 9.1% 39.4% 15.2%	CRYOTHERAPY (*N* = 98)	Always Frequently Sometimes Rarely Never N/A	5.3% 14% 42.1% 15.8% 21% 1.8%	9.1% 18.2% 21.1% 6.1% 36.4% 9.1%
STRETCHING (*N* = 98)	Always Frequently Sometimes Rarely Never N/A	17.5% 33.4% 33.3% 10.5% 5.3% 0%	21.2% 39.4% 39.4% 0% 0% 0%	CONTRAST BATH (*N* = 98)	Always Frequently Sometimes Rarely Never N/A	1.8% 5.3% 28.1% 15.8% 43.8% 5.2%	0% 6.1% 12.1% 21.2% 36.4% 24.2%
ACTIVITY PACING (*N* = 98)	Always Frequently Sometimes Rarely Never N/A	47.4% 35.1% 17.5% 0% 0% 0%	33.3% 45.4% 15.3% 3% 0% 3%	PARAFFIN WAX BATH (*N* = 98)	Always Frequently Sometimes Rarely Never N/A	0% 0% 0% 7% 77.2% 15.8%	0% 0% 3% 9.1% 54.6% 33.3%
ANKLE STRENGTHENING (*N* = 98)	Always Frequently Sometimes Rarely Never N/A	68.4% 19.3% 10.5% 1.8% 0% 0%	39.4% 48.5% 6.1% 3.0% 3.0% 0%	ELECTROTHERAPY (*N* = 98)	Always Frequently Sometimes Rarely Never N/A	1.8% 1.8% 10.5% 15.8% 64.9% 5.7%	9.1% 6.1% 9.1% 12.1% 39.3% 24.3%
HIP STRENGTHENING (*N* = 98)	Always Frequently Sometimes Rarely Never N/A	21.1% 36.8% 31.6%s 10.5% 0% 0%	12.1% 18.2% 27.3% 12.1% 18.2% 12.1%	TAPING (*N* = 98)	Always Frequently Sometimes Rarely Never N/A	1.6% 5.3% 28.1% 29.8% 33.4% 1.8%	9.1% 18.2% 33.3% 27.3% 9.1% 3.%
PROPRIOCEPTION EXERCISES (*N* = 98)	Always Frequently Sometimes Rarely Never N/A	43.9% 43.9% 8.8% 3.4% 0% 0%	24.2% 36.4% 27.3% 0% 9.1% 3%	BRACING (*N* = 98)	Always Frequently Sometimes Rarely Never N/A	1.8% 8.8% 26.3% 33.3% 28.1% 1.7%	6.1% 24.2% 39.4% 21.2% 6.1% 3%
MANUAL THERAPY- LUMBAR SPINE MOBILISATION (*N* = 98)	Always Frequently Sometimes Rarely Never N/A	1.7% 7% 24.6% 33.3% 29.8% 3.6%	3% 6.1% 6.1% 3% 42.4% 39.4%	DRY NEEDLING/ ACUPUNCTURE (*N* = 98)	Always Frequently Sometimes Rarely Never N/A	1.7% 7% 12.3% 10.5% 49.2% 19.3%	0% 0% 15.1% 0% 45.5% 39.4%
CARDIOVASCULAR ENDURANCE EXERCISES (*N* = 98)	Always Frequently Sometimes Rarely Never N/A	12.3% 42.1% 35.1% 8.7% 0% 1.8%	15.2% 36.4% 39.4% 0% 6.2% 3%	CUPPING (*N* = 98)	Always Frequently Sometimes Rarely Never N/A	0% 0% 1.8% 0% 75.4% 22.8%	0% 0% 0% 0% 51.5% 48.5%
GAIT MANAGEMENT (*N* = 98)	Always Frequently Sometimes Rarely Never N/A	31.6% 36.8% 22.8% 7% 0% 1.8%	45.5% 30.3% 15.1% 3% 6.1% 0%	OFF-LOADING (*N* = 98)	Always Frequently Sometimes Rarely Never N/A	3.5% 12.3% 24.6% 36.8% 19.3% 3.5%	3% 6.1% 15.2% 30.3% 36.4% 9%
SELF-MANAGEMENT (*N* = 98)	Always Frequently Sometimes Rarely Never N/A	70.2% 24.6% 5.2% 0% 0% 0%	39.4% 24.2% 18.2% 6.1% 9.1% 3%	PHARMACOLO-GICAL (*N* = 98)	Always Frequently Sometimes Rarely Never N/A	7% 35% 28.1% 12.3% 8.8% 8.8%	9.1% 36.4% 39.4% 9.1% 3% 3%
ORTHOTICS (*N* = 98)	Always Frequently Sometimes Rarely Never N/A	2% 35% 46% 9% 3% 5%	9.1% 63.6% 24.2% 3% 0% 0%	LOCAL ANAETHESIA INJECTION (*N* = 98)	Always Frequently Sometimes Rarely Never N/A	0% 8.77% 7.02% 10.53% 36.84% 36.84%	6.1% 3% 24.2% 9.1% 42.4% 15.2%
VISCO-SUPPLEMENTATION (*N* = 98)	Always Frequently Sometimes Rarely Never N/A	0% 1.7% 5.3% 3.5% 50.9% 38.6%	3% 6% 9% 6% 42% 33%	PLATELET-RICH PLASMA THERAPY (*N* = 98)	Always Frequently Sometimes Rarely Never N/A	0% 0% 1.8% 1.8% 54.4% 42%	3% 0% 3.1% 3% 51.5% 39.4%
STEROID INJECTION (*N* = 98)	Always Frequently Sometimes Rarely Never N/A	1.8% 8.8% 19.2% 22.8% 19.3% 28.1%	9.1% 27.3% 33.3% 6.1% 12.1% 12.1%	SURGERY (*N* = 98)	Always Frequently Sometimes Rarely Never N/A	3.5% 3.5% 35.2% 36.8% 7% 14%	3% 21.2% 57.6% 15.2% 3% 0%

## Discussion

This is the first survey to assess and compare current practice in managing ankle OA amongst physiotherapists and podiatrists in the UK. There is little guidance currently available to help allied healthcare professionals plan an evidence-based management plan of usual care for managing this condition; most guidance is for knee OA and hip OA.

Both professions always or usually used patient education, lifestyle modification, and ankle strengthening exercises, with physiotherapists additionally using self-management and proprioception exercises, whereas podiatrists additionally used activity pacing and gait training. Outside of these five, other commonly used ways to manage ankle OA by physiotherapists were hip strengthening (58%) and by podiatrists were orthotics (73%) and self-management (64%) (Table 2). These treatment choices align with the top three treatment aims in both professions to reduce pain, improve quality of life, and teach self-management. This consensus concurs with many general OA guidelines, such as National Institute for Health and care Excellence (NICE [6], and Osteoarthritis Research Society International (OARSI) which includes patient education, exercise and self-management [7] as core treatment options, although these guidelines are not specific to ankle OA. Additionally, our survey results align with the treatment research agenda from an international foot and ankle OA consortium which recommended understanding usual care of foot and ankle OA to inform the design of control interventions in clinical trials [8]. It is also in line with the modern rehabilitation model for chronic pain which suggests using education to improve the effectiveness of other treatment options, and adding active/self-management techniques as a part of usual care [9].

Some of the treatment options reported by physiotherapists suggest they extrapolated evidence from other joint sites and applied to ankle OA. Hip strengthening was commonly used by physiotherapists (58%) but not podiatrists (27%), which does not align with a systematic review recommending hip strengthening for the conservative management of knee OA but not ankle OA [10]. There were several treatment options which were used sometimes, rarely or never by physiotherapists or podiatrists including hydrotherapy, electrotherapy, dry needling/acupuncture, taping, and intra-articular injections of visco-supplementation or platelet-rich plasma (PRP). Possible reasons include lack of access to hydrotherapy, contradictory OA guidelines by NICE [5] and OARSI [7] about acupuncture for OA, a lack of injection skills, and either the limited availability of visco-supplementation products or PRP in the NHS or a lack of supporting evidence. The observation that few physiotherapists (4%) and podiatrists (15%) used electrotherapy to treat ankle OA contrasts with NICE [5] and OARSI [7] guidelines recommending transcutaneous electrical nerve stimulation (TENS) to alleviate pain. Less than a third of respondents in either professions opted for ankle bracing or taping as usual care. This is surprising, particularly with physiotherapists, since taping is commonly used to reduce ankle range of motion and improve joint stability [11]. The aim of ankle braces is similar [12] but the low uptake of bracing might be due to the survey not specifying the stage of ankle OA clinicians should consider when responding to these questions. It might be that some clinicians only consider ankle bracing in end-stage ankle OA. Orthotics were used significantly more by podiatrists (73%) than physiotherapists (37%), consistent with the finding of a survey of physiotherapists’ and podiatrists’ treatment of plantar fasciitis that foot orthotics were considered the specialist role of podiatrists [13]. It is possible that physiotherapists refer to other services for orthotics but our survey did not distinguish those fitting orthotics themselves and those referring to podiatric or orthotic services. The use of footwear, as opposed to orthotics, was not available as a treatment option in the survey and it is possible that respondents may have opted for this if it had been available. Interestingly, despite podiatrists being asked to contribute at the survey’s development stage, a ‘footwear’ option was not suggested and so was not included in the final version. Although this survey provides data from physiotherapists and podiatrists as to what is current practice for ‘usual care’ in ankle OA, this has not yet been robustly assessed for its efficacy and should be the target for future funding applications.

A survey on the management of foot and ankle OA by GPs in Australia found that pharmacological management was favoured over active and self-management techniques [14]. Our survey found medications were used by nearly half of both physiotherapists and podiatrists. There are approximately 1400 physiotherapists and 450 podiatrists registered as independent or supplementary prescribers in the UK. [15], but we did not ask whether medications were prescribed directly by the physiotherapist or podiatrist, or whether the patient was required to see another healthcare professional to obtain a prescription.

This study had some limitations. Only UK-based practitioners were included, limiting the generalizability of our findings. Our response of 98 is smaller than the 191–293 physiotherapists or podiatrists who responded to electronic surveys of the management of other musculoskeletal conditions [15–18]. It is not possible to establish the exact response to the study owing to its online nature and the denominator who saw the survey invitation being unknown. This study did not identify the length of participants’ clinical experience which might have influenced responses if some treatments such as prescribed medications or intra-articular injections would be more likely be administered by more experienced practitioners. However, just over a third of physiotherapists and half of podiatrists had a master’s degree as their highest qualification which suggests a level of seniority and experience amongst many respondents and the possibility of bias towards these more advanced treatments. We did not make a distinction between foot orthoses, ankle orthoses and ankle foot orthoses (AFOs). Some clinicians might interpret these terms differently from our intended interpretation of an insole type of support. Lastly, due to the small sample sizes in each group, no comparison was made between private and NHS practice, nor in the practice between different countries of the UK.

## Conclusions

The current practice for managing ankle OA amongst physiotherapists and podiatrists has been surveyed in the UK for the first time. The most common treatment aims in both professions were to reduce pain and improve quality of life. The most common treatment programme comprised 3–4 sessions, each lasting 30–40 min. The five most common modalities currently used by physiotherapists were patient education, self-management, lifestyle modification, ankle strengthening, and proprioception exercises. For podiatrists, the five most common were patient education, ankle strengthening, activity pacing, lifestyle modification, and gait training. This study provides a better understanding of how ankle OA is treated in current clinical practice and can inform future clinical trials to assess usual care and compare this with new treatment modalities.

## Data Availability

All datasets used during the current study are available from the corresponding author on reasonable request.

## Abbreviations

OA: Osteoarthritis
UK: United Kingdom of Great Britain and Northern Ireland
GP: General Practitioner
NICE: National Institute of Health and Clinical Excellence
OARSI: Osteoarthritis Research Society International
TENS: Transcutaneous Electrical Nerve Stimulation
PRP: Protein-Rich Plasma
iCSP: Interactive Chartered Society of Physiotherapy
HCPC: Health Care Professions Council
NHS: National Health Service
